# Metabolic engineering of *Rhodopseudomonas palustris* for the obligate reduction of *n*-butyrate to *n*-butanol

**DOI:** 10.1186/s13068-017-0864-3

**Published:** 2017-07-11

**Authors:** Devin F. R. Doud, Eric C. Holmes, Hanno Richter, Bastian Molitor, Georg Jander, Largus T. Angenent

**Affiliations:** 1 Department of Biological and Environmental Engineering, Cornell University, Ithaca, NY 14853 USA; 2 Boyce Thompson Institute for Plant Research, Ithaca, NY 14853 USA; 3 Atkinson Center for a Sustainable Future, Cornell University, Ithaca, NY 14853 USA; 4 Center for Applied Geosciences, University of Tübingen, Hölderlinstr. 12, 72074 Tübingen, Germany

**Keywords:** Metabolic engineering, Redox-driven obligate reduction, *n*-Butanol, *Rhodopseudomonas palustris*

## Abstract

**Background:**

*Rhodopseudomonas palustris* is a versatile microbe that encounters an innate redox imbalance while growing photoheterotrophically with reduced substrates. The resulting excess in reducing equivalents, together with ATP from photosynthesis, could be utilized to drive a wide range of bioconversions. The objective of this study was to genetically modify *R. palustris* to provide a pathway to reduce *n*-butyrate into *n*-butanol for maintaining redox balance.

**Results:**

Here, we constructed and expressed a plasmid-based pathway for *n*-butanol production from *Clostridium acetobutylicum* ATCC 824 in *R. palustris.* We maintained the environmental conditions in such a way that this pathway functioned as the obligate route to re-oxidize excess reducing equivalents, resulting in an innate selection pressure. The engineered strain of *R. palustris* grew under otherwise restrictive redox conditions and achieved concentrations of 1.5 mM *n*-butanol at a production rate of 0.03 g L^−1^ day^−1^ and a selectivity (i.e., products compared to the consumed substrate) of close to 40%. Since the theoretical maximum selectivity is 45%, the engineered strain converted close to its maximum selectivity.

**Conclusions:**

The innate redox imbalance of *R. palustris* can be used to drive the reduction of *n*-butyrate into *n*-butanol after expression of a plasmid-based enzyme from a butanol-producing *Clostridium* strain.

**Electronic supplementary material:**

The online version of this article (doi:10.1186/s13068-017-0864-3) contains supplementary material, which is available to authorized users.

## Background

*Rhodopseudomonas palustris*, which is a member of the purple non-sulfur bacteria group, is often noted for its metabolic versatility. One of these metabolic activities, which is H_2_ production by photofermentation, utilizes organic carbon under anaerobic conditions with light and has become an attractive application for bioenergy production [1]. The ability to produce H_2_ at high concentrations is driven by an excess of reducing equivalents that accumulate during photoheterotrophic growth [2]. Here, we aim to exploit the excess reducing equivalents generated during photoheterotrophic growth to drive an engineered reduction reaction for the obligate production of the liquid fuel *n*-butanol (hereafter butanol).

When growing on acetate, McKinlay and Harwood [1] reported that approximately half of all reducing equivalents that were generated were used for biosynthesis, while the other half required re-oxidation by some alternate pathway such as CO_2_ fixation, H_2_ evolution, or a combination of both. These two pathways were found to be complementary in this role, and when one route was made unavailable, the other facilitated the re-oxidation of all reducing equivalents with minimal changes occurring elsewhere in the central metabolism. However, when fed a more reduced substrate, such as *n*-butyrate (hereafter butyrate), it was observed that under non-H_2_ evolving conditions, *R. palustris* could only grow if exogenous CO_2_ was supplemented into the medium [2, 3]. This finding demonstrates that under these conditions, *R. palustris* generates more reducing equivalents than available electron sinks can accommodate, and ultimately suffers a lethal redox imbalance. Numerous studies have used these principles to improve H_2_ yields from photofermentation using *R. palustris* [4–6].

Recently, Fixen et al. [7] engineered a strain of *R. palustris* to utilize excess reducing equivalents to reduce CO_2_ into CH_4_ by remodeling the nitrogenase enzyme active site and expressing the modified gene from the chromosome. A NifA* strain of *R. palustris* was chosen as the host because it expresses the nitrogenase gene constitutively even in the presence of NH_3_. The result was a light-dependent *R. palustris* strain that produced CH_4_ with ATP coming from cyclic photophosphorylation and electrons coming from either an organic substrate or thiosulfate. This reduction is, however, not obligatory and a large excess of H_2_ was produced by the nitrogenase compared to CH_4_ [7]. Another attractive route to redirect the excess reducing equivalents and to maintain redox balance when growing on butyrate is to produce the fuel butanol through an obligate reduction reaction. The advantage of the obligate reduction is the lack of side products to achieve a high selectivity (i.e., products compared to the consumed substrate).

**Fig. 1:**
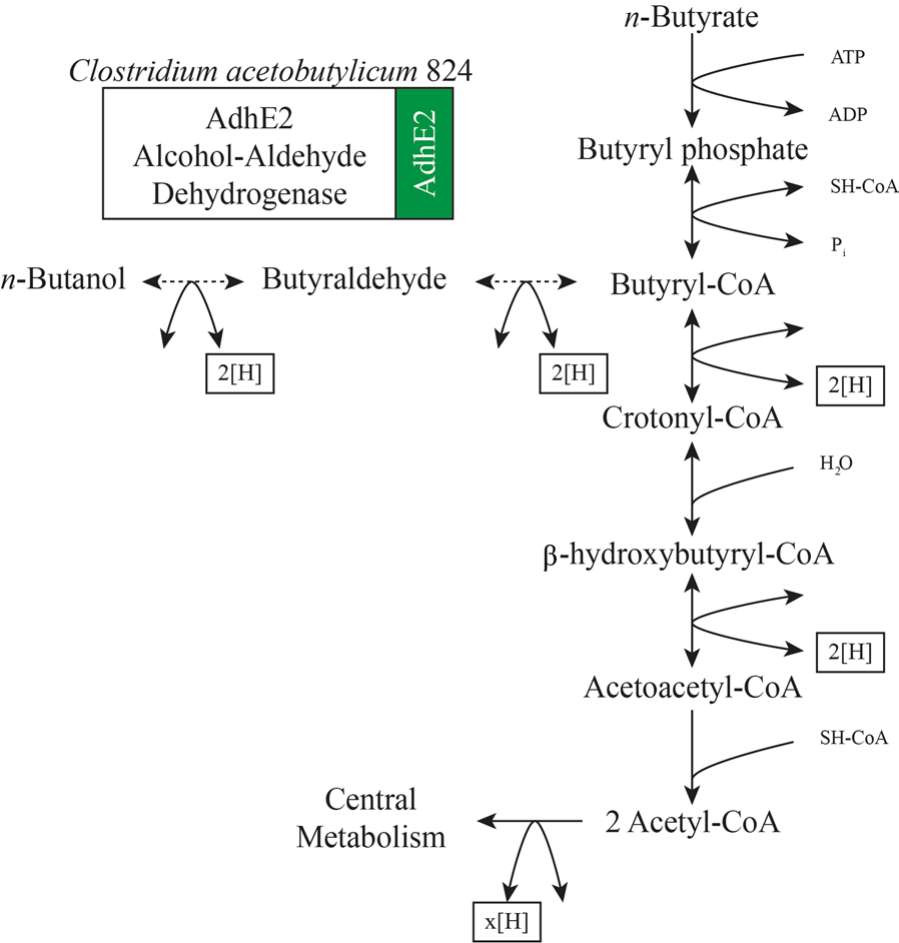
Butyrate metabolism and the proposed route for butanol production in *R. palustris* by the activity of the butyryl-CoA to butanol AdhE2 enzyme. *Dotted arrows* represent the proposed pathway and *solid arrows* indicate native metabolism

Butanol is an attractive biofuel molecule due to its higher energy density, lower volatility, and complete intersolubility with traditional fuels compared to ethanol [8]. Butanol is traditionally produced via fermentation of monosaccharides by certain strains of Clostridia. *Clostridium acetobutylicum* ATCC 824, which has been widely studied for this activity, is generally seen as the model organism for solvent production and previously has been used as a source of genes for engineered butanol production [9–11]. In *C. acetobutylicum*, reduction of butyrate proceeds through a two-phase pathway. First, butyrate is activated to butyryl-CoA, which can occur by two different routes: (1) a two-step butyrate kinase/phosphotransbutyrylase reaction (Fig. 1); or (2) a one-step reaction with butyrate/acetoacetyl-CoA transferase transferring a CoA group from acetoacetyl-CoA to butyrate. Second, butyryl-CoA is reduced to butanol through the butyraldehyde intermediate [12, 13]. The AdhE2 enzyme from *C. acetobutylicum* ATCC 824 (hereafter AdhE2_824_ and *adhE2*_824_ for its gene) reduces butyryl-CoA to butyraldehyde and subsequently butanol (Fig. 1) [14]. Analysis of AdhE2_824_ has revealed that the protein is a bifunctional NADH-dependent fusion protein with the N-terminal domain retaining highly conserved sequences of the aldehyde dehydrogenase family and the C-terminal domain retaining conserved sequences of the iron-containing alcohol dehydrogenase family [15]. A second alcohol dehydrogenase enzyme BdhB has also been found to aid in the reduction of butyraldehyde to butanol in *C. acetobutylicum*, though its activity is not necessary for butanol production [16].

In planning to introduce the butanol production pathway together with the existing catabolism of butyrate by *R. palustris*, we established that before butyrate can be converted to two molecules of acetyl-CoA via anaerobic β-oxidation, butyryl-CoA is first formed through native butyrate kinase/phosphotransbutyrylase enzymes (Fig. 1). Under photoheterotrophic redox imbalance, the first oxidation reaction for the metabolism of butyrate (i.e., butyryl-CoA to crotonyl-CoA in Fig. 1) would likely be inhibited by the thermodynamic limitations of the reduced cofactor pool. Because of this, we anticipated the butyryl-CoA intermediate to accumulate and provide an ideal target for shunting excess reducing equivalents for the production of butanol by introducing only *adhE2*_824_ (the bifunctional aldehyde/alcohol dehydrogenase gene) into *R. palustris* (Fig. 1). After necessary optimizations, the production of butanol indicated that this assumption was correct.

A direct introduction of plasmid-based *adhE2*_824_ did not result in the production of AdhE2_824_ (and butanol production) due to the major incompatibilities in codon utilization preference between the *C. acetobutylicum* source and the *R. palustris* host. Therefore, we pursued two alternate attempts to introduce the activity of AdhE2 by using: (1) a gene homologous to *adhE2*_824_ from *R. palustris* strain BisB18 (hereafter *adhE*_BisB18_ and AdhE_BisB18_ for its protein); and (2) a codon-optimized *adhE2*_824_ (hereafter *adhE2*_opti_ and AdhE2_opti_ for its protein). In all our growth experiments, we omitted HCO_3_^−^ (CO_2_) from, and added NH_3_ to, the growth medium to block the normal routes of shunting reducing equivalents, resulting in an obligate butyrate reduction. This strong selection pressure will only allow growth when butanol production occurs. In our hands, butanol production through the homologous AdhE_BisB18_ did not work, but AdhE2_opti_ enabled recovered growth through butanol production. Subsequently, we were able to increase the butanol selectivity and production rate in further experiments, accomplishing our aim of generating an engineered reduction reaction for the obligate production of the liquid fuel butanol in *R. palustris*.

## Methods

### Media and culture conditions

*Rhodopseudomonas palustris* CGA009 was cultured in anaerobic butyrate Rhodospirillaceae medium (RM) [17], consisting of 1.64 g NaHCO_3_, 0.5 g KH_2_PO_4_, 0.5 g K_2_HPO_4_, 0.2 g MgSO_4_·7 H_2_O, 0.4 g NaCl, 0.05 g CaCl_2_·2 H_2_O, 0.01 g Fe-Citrate, 1.0 g (NH_4_)_2_SO_4_, 1.12 mL 10 M NaOH, and 1 mL trace metals per liter. The trace-metal solution consisted of 0.25 mL concentrated HCl, 70.0 mg ZnCl_2_, 100.0 mg MnCl_2_·4 H_2_O, 60.0 mg H_3_BO_3_, 200.0 mg CoCl_2_·6 H_2_O, 20.0 mg CuCl_2_·2 H_2_O, 20.0 mg NiCl_2_·6 H_2_O, and 40.0 mg Na_2_MoO_4_·2 H_2_O L^−1^. After autoclaving, 1.0 g sterile butyric acid was added as the substrate. No toxicity or inhibition of *R. palustris* growth was detected at this concentration (Additional file 1). 1 mL trace vitamins was added to the medium. The trace-vitamin solution consisted of 1.0 g *p*-aminobenzoic acid, 1.0 g thiamine, and 0.1 g biotin L^−1^. Sterile medium (50 mL) with a final pH of 7.0 was added to autoclaved serum bottles and sparged for 20 min with sterile-filtered 80:20 N_2_/CO_2_ gas. For the growth of engineered strains under restrictive butyrate-to-butanol conversion conditions, NaHCO_3_ was omitted from the medium, the addition of 10 M NaOH was increased to 2.3 mL total, and serum bottles were sparged for 20 min with N_2_ instead of N_2_/CO_2_. In addition, 200 μg mL^−1^ of kanamycin sulfate was added for retention of the plasmid in all growth conditions with engineered strains. All serum bottle cultures were grown in an environmental growth chamber (GC8-2VH, EGC, Chagrin Falls, OH) at 30 °C with 80 μmol of photosynthetically active photons s^−1^ m^−2^ (photons between 400 and 700 nm) illumination from both fluorescent and incandescent lamps.

### DNA manipulation and cloning

We used the green fluorescence protein GFPmut2 [18] as a reporter protein to screen for the activity of different expression vectors in *R. palustris* under aerobic conditions. We tested two different plasmids as candidate expression vectors: (1) the endogenously derived pMG105 [19]; and (2) the broad-host pBBR1MCS-2 [20]. For detection by immunoreaction, we introduced a C-terminal 6X His tag *prior* to the termination site with PCR by the reverse primer (Table 1). *GFPmut2* was inserted into the pMG105 and pBBR1MCS-2 vectors under the control of a range of promoters, including the endogenous phosphoenolpyruvate carboxykinase (*ppckA*) from *R. palustris*, arabinose (*ara*), and lactose (*lac*) promoters. The *adhE*_BisB18_ and *adhE2*_824_ genes were amplified from *R. palustris* strain BisB18 and *C. acetobutylicum* ATCC 824, respectively, using Phusion HF polymerase (New England Biolabs, Ipswich, MA) and directionally ligated into the pBBR1MCS-2 expression vector using the XhoI/XbaI cloning sites. Restriction and ligation enzymes were purchased from Promega (Fitchburg, WI) and all constructs were verified with Sanger sequencing (Cornell University Genomics Facility, Ithaca, NY). *Escherichia coli* DH5α was used for routine cloning of constructs. Both *E. coli* and *R. palustris* were transformed by electroporation with a Bio-Rad Gene Pulser II Electroporator (Bio-Rad, Hercules, CA) with 1.8 kV, 200 Ω, 25 µF for *E. coli*, and 2.5 kV, 400 Ω, 25 µF for *R. palustris*. Electroporated cells were recovered in SOC for 1 and 3 h before plating on lysogeny broth (LB) agar plates with 50 and 200 μg mL^−1^ kanamycin sulfate for isolation of individual *E. coli* and *R. palustris* transformants, respectively.

**Table 1:** Sequences of primers used for this study

Primer	Length	Sequence (5′ → 3′)	Purpose
pBBR1MCS-2 vector			
XhoI GFPmut2 F1	60	GGCGGCCTCGAGAGGAGGATCTATTCATGAGTAAAGGAGAAGAACTTTTCACTGGAGTTG	Cloning *GFPmut2*
XbaI GFPmut2 R1	51	GCCGCCTCTAGACTATTTGTATAGTTCATCCATGCCATGTGTAATCCCAGC	
BamHI RBS AdhE2_824_ F1	68	GCCGCGGATCCAGGAGGATCTATTCATGAAAGTTACAAATCAAAAAGAACTAAAACAAAAGCTAAATG	Cloning *adhE2*_824_
SacI 6X His AdhE2_824_ R1	62	CACCCGGAGCTCTAAGTGGTGATGGTGATGATGAAATGATTTTATATAGATATCCTTAAGTT	
SacI AdhE2_824_ R1	43	CACCCGGAGCTCTAAAATGATTTTATATAGATATCCTTAAGTT	
XhoI RBS AdhE_BisB18_ F1	57	GACGACCTCGAGAGGAGGATCTATTCGTGACCTTATCTACCCCGTCCGACCTCGACA	Cloning *adhE*_BisB18_
XbaI 6X His AdhE_BisB18_ R1	60	CACCCGTCTAGACTAGTGGTGATGGTGATGATGTTCCGCGGCGTTCGCCGTCGCCACCGA	
XbaI AdhE_BisB18_ R1	52	CAGTCACCCGTCTAGACTATTCCGCGGCGTTCGCCGTCGCCACCGACAATGT	
pMG105P vector			
XbaI GFPmut2 F1	60	GGCGGCTCTAGAAGGAGGATCTATTCATGAGTAAAGGAGAAGAACTTTTCACTGGAGTTG	Cloning *GFPmut2*
SalI GFPmut2 R1	53	GGCGGCCGGTCGACCTATTTGTATAGTTCATCCATGCCATGTGTAATCCCAGC	

Restriction sites are underlined

To successfully produce AdhE2_824_ in *R. palustris*, codon optimization was pursued. Optimization design was performed in-house and largely utilized the one amino acid one codon approach, except in cases of multiple codon repeats and unfavorable energetics of mRNA secondary structure (Additional file 2). *adhE2*_opti_ with a C-terminal 6X His tag was synthesized by GenScript (Piscataway, NJ) with flanking XhoI/XbaI restriction sites that were used for directional cloning into pBBR1MCS-2.

### SDS-PAGE and western blot detection

For both SDS-PAGE and subsequent western blot detection, 50 mL of late-log *E. coli* and *R. palustris* cells were harvested by centrifugation (10 min, 12,000×*g*, 4 °C) and resuspended in 1 mL 1X Laemmli sample buffer [21] with a protease inhibitor (Protease inhibitor cocktail, Promega, Fitchburg, WI). The suspension was lysed by ultrasonication on ice using three 30 s on 1 min off pulses of 20 W (Branson Sonifier 150, Emerson, Danbury, CT). After sonication, debris was removed by centrifugation and the total soluble protein concentration was determined by BCA assay (Pierce BCA protein assay kit, Thermo Scientific, Rockford, IL). Forty-five μL of 3 mg mL^−1^ soluble protein samples and 10 μL of Precision Plus Protein™ standard (Bio-Rad, Hercules, CA) were loaded into pockets of a Mini-PROTEAN^®^ TGX gel (Bio-Rad, Hercules, CA) and run at 100 V for 1.5 h before blotting onto PVDF membranes at 100 V for 1 h. For the initial western blot, immunoreactive bands were developed using manufacturer-suggested protocols for the 1 h Western™ Standard Kit for rabbit (GenScript, Piscataway, NJ) and a 6X His primary antibody (Rabbit polyclonal, GeneTex, Irvine, CA). We used a similar protocol a second time to improve the sensitivity with these changes: we used 0.1 mL OD_600_ 1.0 cells with 0.1 mL Laemmli sample buffer. The samples were boiled for 10 min before centrifugation. 15 µL of each sample was loaded into separate gel lanes. For development, an enhanced chemiluminescence protocol was used using the manufacturer-suggested protocol for promega ECL western blotting substrate (Fitchburg, WI). The film was allowed to incubate with the PVDF membrane for 30 min *prior* to development.

### Bioreactor configuration and operation

*Prior* to operation, sealed glass bioreactors were autoclaved and filled with 350 mL sterile RM with 1 g L^−1^ butyrate and without HCO_3_^−^. The bioreactors were equipped with a recirculating water jacket that was operated at 30 °C. Magnetic stir plates (IKA Works Inc., Wilmington, NC) provided stirring of the reactors at 450 rpm. The bioreactors were continuously sparged with sterile-filtered N_2_ gas at a flow rate of 0.1 L min^−1^ to maintain an anaerobic environment. After sparging for 1 h to ensure anaerobic reactor conditions, 1 mL of actively growing *R. palustris* cells at an OD_600_ of 0.1 was inoculated into the bioreactor. The preculture of *R. palustris* pBBR1MCS-2 *lacp adhE2*_opti_ was grown in anaerobic RM without HCO_3_^−^, while the preculture of *R. palustris* pBBR1MCS-2 *lacp GFPmut2* (control) was grown in aerobic RM without HCO_3_^−^. Gas entered the liquid phase of each bioreactor through a sparging stone to increase the gas/liquid interface for butanol stripping. Off-gas was connected to 2 Friedrich’s condensers in parallel with recirculating water at 5 °C to condense the butanol that had been removed by gas stripping. Gas was released into the atmosphere after one passing through the condensers. The bioreactor volume was illuminated externally with a single 60-W incandescent lamp as used previously [22]. The bioreactors were operated in batch-mode and liquid samples were periodically taken for OD_600_ and metabolite measurements.

### Metabolite detection

Butyrate, butanol, and acetate were measured via HPLC (600 HPLC, Waters, Milford, MA) with a refractive index detector and an Aminex HPX-87H column (Bio-Rad, Hercules, CA). The column temperature was set to 60 °C, and a 5-mM sulfuric acid eluent at a flow rate of 0.6 mL min^−1^ was used as the mobile phase. Butanol and butyraldehyde were measured with a gas chromatograph (HP 5890, Hewlett Packard, Palo Alto, CA) with a 7673 autoinjector and flame ionization detector. The gas chromatograph contained a custom-made packed bed glass column of 1.8 m × 2 mm id (Supelco, Sigma-Aldrich, St. Louis, MO). The support matrix of the column was Chromosorb W/AW80 over 100 mesh; phases were preconditioned: phase A was 10% Carbowax-20M; phase B was 0.1% phosphoric acid. The inlet and detector temperatures were 220 and 240 °C, respectively. The column temperature profile was 100 °C for 2 min, a ramp of 40 °C min^−1^ to 180 °C with a 5-min hold. Butanol concentrations were verified by both HPLC and GC platforms.

## Results and discussion

### GFP screen of expression vectors in *R. palustris* CGA009

**Fig. 2:**
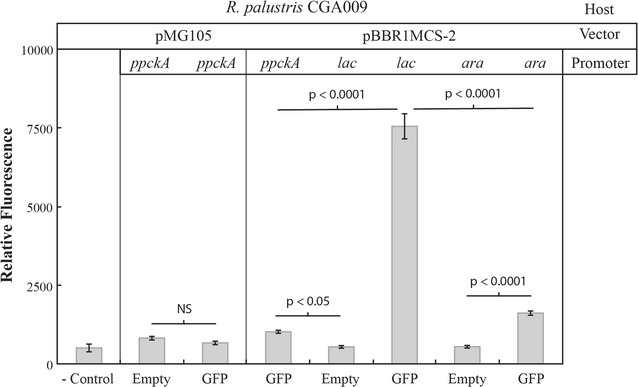
Fluorescence screen of potential expression vectors in *R. palustris* CGA009. Fluorescence from each strain is expressed as relative fluorescence per 1 unit of OD_600_ in an exponentially growing culture. Technical triplicates with standard deviation and relevant* p* values are shown. Excitation/emission was measured at 480:510 nm. The control contained no cells. Different empty vectors and constructs with *GFPmut2* are designated by vector/promoter heading

Different combinations of promoter and vector sequences were screened for highest levels of expression within *R. palustris*. The endogenous *ppckA* promoter of *R. palustris* is one of the few promoter sequences that had been previously validated to be active in *R. palustris* under gluconeogenic conditions [23]. However, we only observed a minimal increase in fluorescence for both the pMG105 and pBBR1MCS-2 *ppckAp GFPmut2* constructs compared to the empty vector controls (Fig. 2). While the pBBR1MCS-2 *arap GFPmut2* construct yielded slightly higher levels compared to *ppckAp*, the pBBR1MCS-2 *lacp GFPmut2* construct offered the best expression overall and was selected as the vector for all further expressions in *R. palustris* (Fig. 2). Expression from the *lac* promoter in *R. palustris* was not affected by the addition of the inducer IPTG and catabolite repression only showed a minor decrease in fluorescence (Additional file 3). This suggests that the *lac* regulatory response system utilized by *E. coli* is not functional in *R. palustris*. Although the pBBR1MCS-2 *lac* promoter system provided the best expression in *R. palustris* of all the combinations tested (Fig. 2), the fluorescent signal was still ~13 times lower than the signal from *E. coli* pBBR1MCS *lacp GFPmut2* (data not shown).

### Engineering *adhE*_BisB18_ in *R. palustris*

**Fig. 3:**
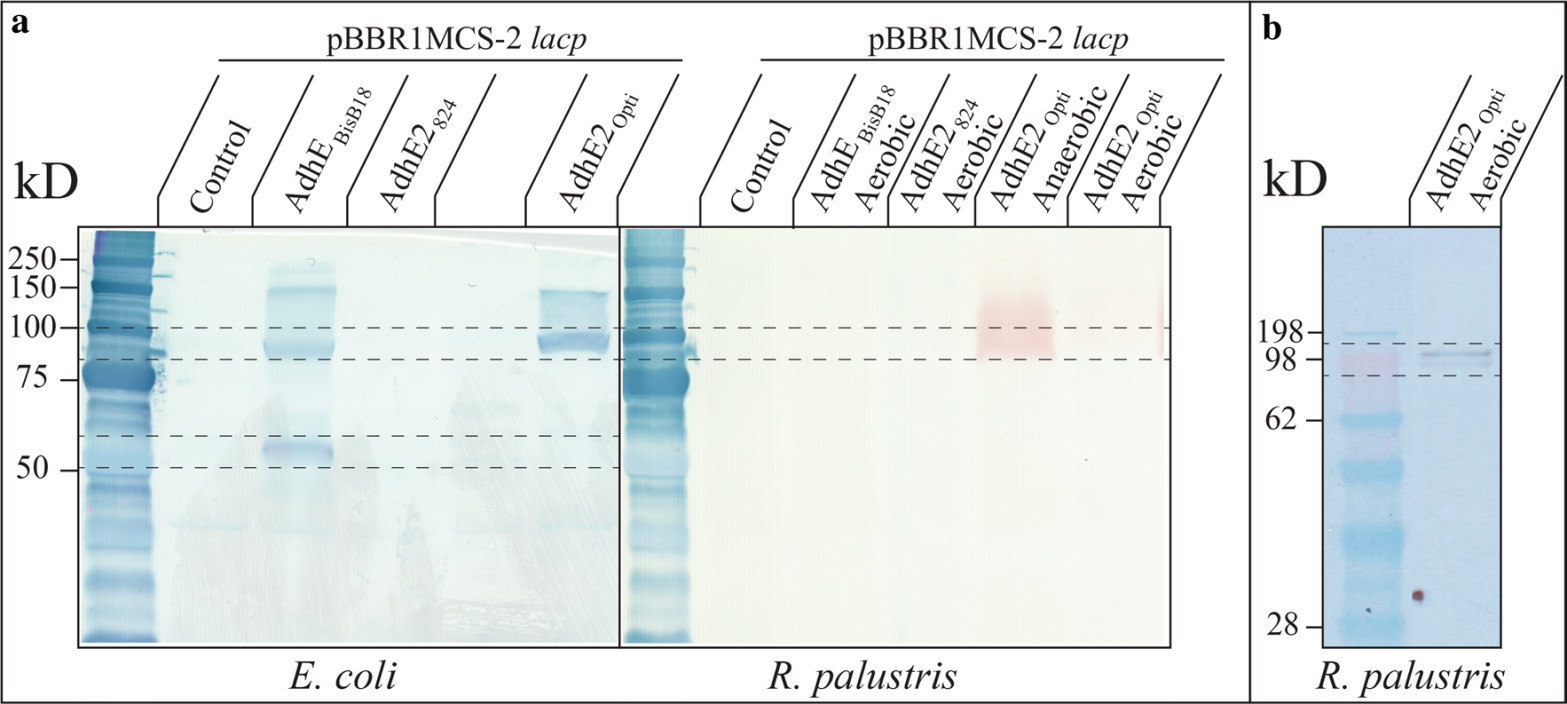
Western blots for the detection of AdhE2 via the C-terminal 6X His tag: **a** in both *E. coli* and *R. palustris*. Expected AdhE_BisB18_, AdhE2_824_, and AdhE2_opti_ sizes are 94.7, 94.3, and 94.3 kD, respectively (*outlined with dotted box*). Downfield band of ~50 kD (*outlined with dotted box*) likely corresponds to the fragmented C-terminal alcohol dehydrogenase domain where the 6X His tag is located. Protein amounts of 135 μg were loaded into all sample lanes. The *red* band in the *R. palustris* anaerobic *lane* is from photo pigments and may partially be obscuring signal from the western blot: **b** in *R. palustris* pBBR1MCS-2 *lacp adhE2*_opti_ cultures. The signal size of aerobic cultures of *R. palustris* pBBR1MCS-2 *lacp adhE2*_opti_ corresponded with the expected size of AdhE2_opti_ (94.3 kD)

As determined by the lack of growth under restrictive conditions, the absence of butanol production (data not shown), and the lack of a signal in a western blot under permissive conditions (Fig. 3a, AdhE_BisB18_ Aerobic), direct introduction of *adhE2*_824_ in the pBBR1MCS-2 *lacp* construct did not result in the production of AdhE2_824_ in *R. palustris*. This is likely due to the large GC discrepancy (30% vs. 65%) and codon utilization preference between *C. acetobutylicum* and *R. palustris* [24]. To address this, we looked for homologous proteins within *R. palustris*. Indeed, endogenous alcohol/aldehyde dehydrogenases with homology to AdhE2_824_ have been found to function in the production of butanol from butyrate when overexpressed. This was previously demonstrated with the *fucO* gene in *E. coli*, where overexpression allowed production of butanol from an engineered reverse β-oxidation pathway where no butanol production was observed previously [25]. In an attempt to enable butanol production in *R. palustris*, a BLAST search for an endogenous enzyme yielded the identification of RPC_4481 (AdhE_BisB18_) in *R. palustris* strain BisB18, which is an aldehyde/alcohol dehydrogenase with high homology to both domains of AdhE2_824_.

Before expressing *adhE*_BisB18_ as a candidate butanol-pathway gene, *R. palustris* strain BisB18 was screened for its ability to grow by producing butanol from butyrate under restrictive electron sink conditions (without HCO_3_^−^). Although no growth or butanol production occurred (data not shown), it is possible that wild-type production of this protein under these conditions is either inactive or insufficient for an observable effect, similar to the FucO protein in *E. coli* [25]. Because no experimental studies have previously characterized the activity of AdhE_BisB18_, its highly conserved primary sequence was further investigated for its structural homology to the verified butanol-producing AdhE2_824_.

Using Swiss-Pdbviewer/DeepView [26], three-dimensional structures of the individual protein domains for both AdhE2_824_ and AdhE_BisB18_ were generated. The predicted tertiary structure of both aldehyde/alcohol dehydrogenases appeared highly conserved with regard to overall shape, location, active site residues, channel volume, and surface charge (Additional file 4). This suggested that the two proteins likely recognized similar substrates. We, therefore, expressed *adhE*_BisB18_ in *R. palustris* using the pBBR1MCS-2 *lacp* construct. Transformants were inoculated into RM with butyrate and with and without HCO_3_^−^ to screen for viability and butanol production, respectively. Although transformants grew up with HCO_3_^−^, no growth was observed without HCO_3_^−^ condition (data not shown), suggesting that either AdhE_BisB18_ was not successfully produced by *R. palustris* CGA009 or did not have butyryl-CoA-to-butanol conversion activity.

To investigate this further, a crude cell extract of the culture with HCO_3_^−^, along with *E. coli* harboring the same construct, was analyzed via western blotting. Although the strong signal at the expected size of 94.5 kD for AdhE_BisB18_ for *E. coli* pBBR1MCS-2 *lacp adhE*_BisB18_ demonstrated that the protein was produced (Fig. 3a, AdhE_BisB18_), the lack of a signal from *R. palustris* pBBR1MCS-2 *lacp adhE*_BisB18_ suggested that the protein was either not produced or was produced at levels below detection (Fig. 3a, AdhE_BisB18_ Aerobic). Aerobic conditions were preferred since anaerobic cultures often had photosynthetic pigments overlapping the expected size for AdhE (Fig. 3a, AdhE2_opti_ Anaerobic). The lack of growth and butanol production under restrictive conditions, however, demonstrated conclusively that *R. palustris* pBBR1MCS-2 *lacp adhE*_BisB18_ was not endowed with butyrate-to-butanol conversion functionality (data not shown).

### Engineering *adhE2*_opti_ in *R. palustris*

Another route to circumvent the large GC discrepancy and codon utilization preference between *C. acetobutylicum* and *R. palustri*s was to perform codon optimization on *adhE2*_824_ to enable expression. The codon utilization table for *R. palustris* CGA009 was determined by analysis of all genomic coding sequences and was retrieved from the Kazusa database [27]. The *adhE2*_opti_ gene resulted in an increase of the average codon utilization frequency in *R. palustris* from 23% for the original *adhE2*_824_ to 64% for the optimized *adhE2*_opti_ (Additional file 5). Codon optimization resulted in a shift of the GC content from 32.5% for *adhE2*_824_ to 62% for *adhE2*_opti_. In addition, the presence of rare codons (<10% utilization frequency), which previously composed 47.4% of all codons in *adhE2*_824_, was entirely eliminated. One disadvantage to this approach of codon optimization is that rare codons are known to induce ribosomal pausing, which has been demonstrated important for proper folding of proteins. Thus, with the elimination of all rare codons from *adhE2*_opti_, the elimination of codon-dependent ribosomal pausing could lead to improper folding and aggregation of the target protein [28]. For comparison with *adhE2*_opti_, the codon utilization frequency of *adhE*_BisB18_ averaged 55% (Additional file 5), the gene possessed an overall 63% GC content, and the frequency of rare codons within the gene was 5%, demonstrating the utilization of rare codons for proper translation of proteins in endogenous alcohol/aldehyde genes (Additional file 6).

To test whether *adhE2*_opti_ would be expressed in *R. palustris* CGA009 and enable growth by conversion of butyrate to butanol, the gene was introduced using the pBBR1MCS-2 *lacp* construct. Transformants were then inoculated into RM with butyrate and with and without HCO_3_^−^ for growth screening and western blot analysis. This engineered strain was able to grow without HCO_3_^−^ (restrictive conditions) and it produced a measureable amount of butanol, demonstrating the successful production of AdhE2_opti_ with a functional activity. While a detectable signal was observed on a western blot for the *E. coli* cloning culture, no signal was initially detected from *R. palustris* pBBR1MCS-2 *lacp adhE2*_opti_ (Fig. 3a, AdhE2_opti_ Anaerobic and AdhE2_opti_ Aerobic). We performed a second western blot using a more sensitive X-ray film imaging method. For aerobically grown *R. palustris* pBBR1MCS-2 *lacp adhE2*_opti_, we observed a single signal at approximately 94 kD, demonstrating successful expression (Fig. 3b, AdhE2_opti_ Aerobic). As stated above, GFPmut2 production from the pBBR1MCS *lac* promoter is much lower in *R. palustris* than in *E.* coli, so the lack of a signal in the majority of the samples is likely due to low protein production in *R. palustris*.

### Butanol production rates and selectivity for *R. palustris* pBBR1MCS-2 *lacp adhE2*_opti_

**Fig. 4:**
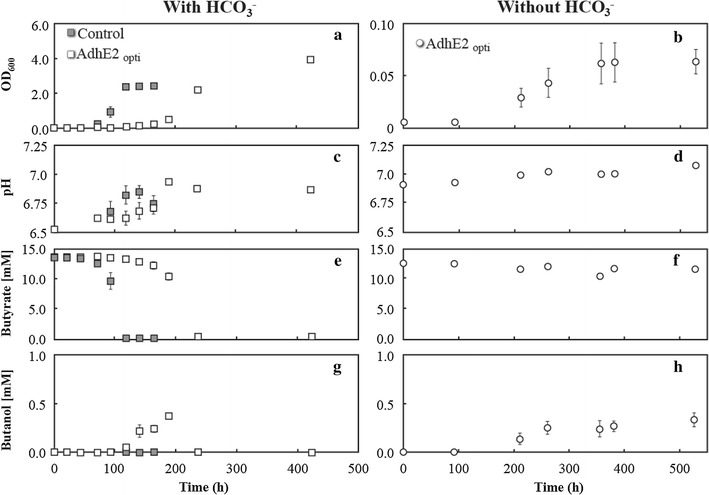
Performance of *R. palustris* pBBR1MCS-2 *lacp GFPmut2* (control) and *R. palustris* pBBR1MCS-2 *lacp adhE2*_opti_ in permissive conditions (with HCO_3_^−^, squares), and *R. palustris* pBBR1MCS-2 *lacp adhE2*_opti_ in restrictive conditions (without HCO_3_^−^, circles): **a**, **b** growth; **c**, **d** pH; **e**, **f** concentration of butyrate in the broth; **g**, **h** butanol concentration in the broth. The results from the control and *R. palustris* pBBR1MCS-2 *lacp adhE2*_opti_ are represented by *gray* and *white* symbols, respectively. Average and standard deviation of biological triplicates are shown

Because of the rescued growth and butanol production observed, *R. palustris* pBBR1MCS-2 *lacp adhE2*_opti_ was further investigated for its ability to grow under both permissive and restrictive conditions and these results were compared to *R. palustris* pBBR1MCS-2 *lacp GFPmut2* as a control. *R. palustris* pBBR1MCS-2 *lacp adhE2*_opti_ was capable of growth without HCO_3_^−^ (restrictive conditions) (Fig. 4b), while this growth did not occur for the control strain. The growth for *R. palustris* pBBR1MCS-2 *lacp adhE2*_opti_ occurred on a long time scale and reached a relatively low maximum cell density of ~0.06 OD_600_ (Fig. 4b) at neutral pH levels (Fig. 4d). Following the onset of the stationary phase, consumption of butyrate and production of butanol still occurred (Fig. 4f, h), but the cultures only reached a maximum butanol concentration of <0.4 mM after extended incubation (Fig. 4h).

Although only low levels of butyrate were consumed (Fig. 4f), butanol production occurred with a 39.7 ± 7.6% selectivity based on moles of carbon. The theoretical maximum for butanol production is based on all electrons derived from complete oxidation of one molecule of butyrate reducing five molecules of butyrate to butanol (6 butyrate → 5 butanol + 4 CO_2_) for 83% conversion efficiency. However, based on previous empirical measurements of excess reducing equivalents of *R. palustris* growing with butyrate by McKinlay et al. [3], only an estimated 0.45 mol of butanol should be produced per mole of butyrate consumed for an effective 45% selectivity (6 butyrate → 2.7 butanol + biomass). Therefore, almost all reducing equivalents predicted to be in excess were being converted into butanol by *R. palustris* pBBR1MCS-2 *lacp adhE2*_opti_ (40% vs. 45%), which we had anticipated for an obligatory reaction.

To further investigate why *R. palustris* pBBR1MCS-2 *lacp adhE2*_opti_ showed low growth rates, *R. palustris* was screened for product (butanol) toxicity. However, no inhibition of growth was observed in *R. palustris* at butanol concentrations up to ~30 mM (Additional file 7), removing our concerns about a concentration of ~0.4 mM (Fig. 4h). In addition, we operated a bioreactor with product removal to investigate whether this would increase growth and butanol production rates by removing butanol from the substrate pool, thereby, improving the thermodynamics for butanol formation and eliminating any end-product inhibition. Sparging of N_2_ has been previously used to remove butanol from fermentation broths [29]. Here, N_2_ gas was continuously sparged into 350-mL anaerobic bioreactors to remove butanol produced by *R. palustris* pBBR1MCS-2 *lacp adhE2*_opti_ in batch-mode for 6 consecutive weeks. The *R. palustris* pBBR1MCS-2 *lacp adhE2*_opti_ culture grew for that entire period up to an OD_600_ of 0.12 (Additional file 8: Fig. S6A), and achieved an average maximum butanol production rate of 0.0017 g L^−1^ day^−1^ (Additional file 8: Fig. S6D) with a maximum butanol concentration of 0.35 mM (Additional file 8: Fig. S6C) without depleting butyrate (Additional file 8: Fig. S6B). The lack of a considerable improvement compared to the earlier experiment showed that neither butanol toxicity nor thermodynamics nor end-product inhibition was a main reason for low growth rates. Finally, we found that the extraction system removed a majority of the butanol without removing any of the butyrate (Additional file 8: Fig. S6E, F).

The intermediate butyraldehyde within microbial cells is another metabolite that could have inhibited *R. palustris*, even though the maximum concentration in the supernatant was measured to be only 50 μM. However, due to the fusion nature of AdhE2_opti_, we anticipate that butyraldehyde would rapidly be converted into butanol with a high flux rate, resulting in a low effective concentration [30]. Without butanol and butyraldehyde toxicity, we can only speculate about why growth and butanol production rates of our engineered strain are so low. Since AdhE2_opti_ is provided as the sole route to maintaining redox balance, all excess electrons must be funneled through this pathway. When the capacity of the AdhE2_opti_ pool is outweighed by the demand for regenerating oxidized reducing equivalents, this would introduce a bottleneck in the maximum rate of metabolism and growth. However, more work is required to be conclusive.

Some butanol production did also occur for *R. palustris* pBBR1MCS-2 *lacp adhE2*_opti_ with HCO_3_^−^ (permissive conditions) when exogenous CO_2_ was available for maintaining redox balance (Fig. 4a, g) at a neutral pH level (Fig. 4c). Because the preculture had originally been maintained without HCO_3_^−^, a long lag phase and carryover of butanol producing activity was observed for *R. palustris* pBBR1MCS-2 *lacp adhE2*_opti_ (Fig. 4a), resulting in a maximum concentration of 0.37 mM with 11.5 ± 2.4% selectivity (Fig. 4e, g). Though the transient production of butanol was a surprise, this lower selectivity for the permissive condition was expected because more reducing equivalents could be used in central metabolism following the introduction of exogenous HCO_3_^−^, resulting in much higher growth (~10× OD at 200 h), the complete depletion of butyrate (Fig. 4e), no production of acetate, and ultimate consumption of all butanol produced (Fig. 4g). This contrasts with the restrictive condition where all growth proceeds through the obligate engineered pathway, butyrate is not depleted, and all butanol produced remains in solution.

### Optimizing butanol production by pregrowing *R. palustris* pBBR1MCS-2 *lacp adhE2*_opti_

**Fig. 5:**
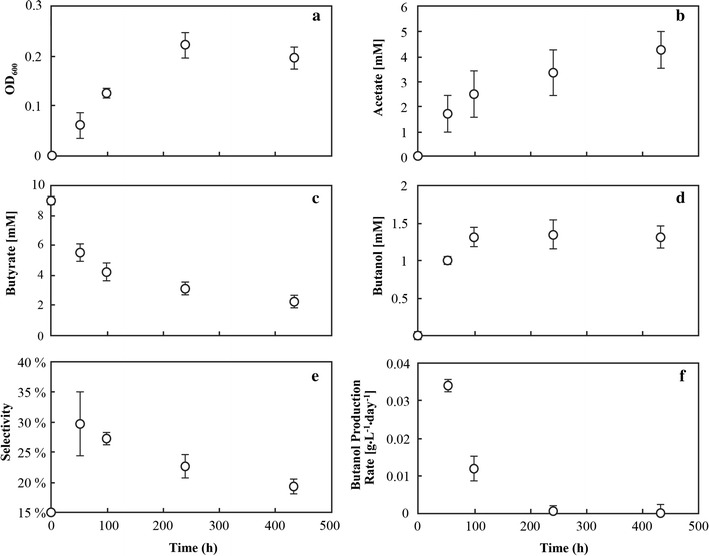
Performance of *R. palustris* pBBR1MCS-2 *lacp adhE2*_opti_ without HCO_3_^−^ in bottles that were inoculated with concentrated cultures: **a** growth with corrected OD_600_ (initial OD_600_ was subtracted); **b** acetate concentration in the broth; **c** butyrate concentration in the broth; **d** butanol concentration in the broth; **e** butanol selectivity; **f** volumetric butanol production rate. *Error bars* show the standard error of three biological replicates

In an attempt to improve the maximum butanol concentrations produced by the engineered strain, 50 mL aerobic cultures was grown to mid-log phase, washed 3 times with sterile RM, resuspended in 0.5 mL RM, and used as an inoculum into 20-mL anaerobic cultures under restrictive conditions. This procedure ensured that the butyrate-to-butanol conversion was not catalyst limited as the initial OD_600_ of these cultures was >1. By using a concentrated inoculum, *R. palustris* pBBR1MCS-2 *lacp adhE2*_opti_ produced butanol concentrations of greater than 1.5 mM at volumetric butanol production rates of 0.034 g L^−1^ day^−1^ (Fig. 5d, f). All butanol was produced within 100 h of inoculation (Fig. 5d, f). Following 100 h, however, the *R. palustris* pBBR1MCS-2 *lacp adhE2*_opti_ culture continued to grow and consume butyrate (Fig. 5a, c) with the main carbon product switching from butanol to acetate (Fig. 5b). Acetate had not been detected before in any of the previous anaerobic experiments and in terms of maintaining redox balance, acetate is an unfavorable redox product compared to butanol. This metabolism reduced the butanol selectivity (Fig. 5e). We do not know exactly why this switch occurred, but it was likely a result from having a high biomass culture that underwent a rapid switch from aerobic to anaerobic conditions.

## Conclusion

The introduction of *adhE2*_opti_ into *R. palustris* via a plasmid-based construct is an example of metabolically engineered fuel production that proceeds as an obligate strategy for growth. Although growth and production rates were low for this system, our selectivity of 39.7 ± 7.6% matched well with the empirical expectation of 45.0% under these conditions. Because the low growth rates and butanol production are likely constrained by redox flux through the engineered pathway, rational design and directed evolution could be used to further improve the performance of this activity. If improved, the system could be easily integrated into a simple bioreactor design with highly efficient selectivity and recovery. While this proof-of-concept for harnessing an obligate reducing metabolism to drive engineered reactions used butanol as a target molecule, this redox-based driving force could be used to achieve other reactions for the production of desirable chemicals that require reduction reactions.

## Additional files

**Additional file 1.** Growth with butyrate, containing Figure S1.

**Additional file 2.** Sequence.

**Additional file 3.** Fluorescence, containing Figure S2.

**Additional file 4.** Structure, containing Figure S3.

**Additional file 5.** Codons, containing Figure S4.

**Additional file 6.** Codons and GC content, containing Table S1.

**Additional file 7.** Growth with butanol, containing Figure S5.

**Additional file 8.** Growth with product removal, containing Figure S6.
