# Supplementary material for: Metabolic engineering of Rhodopseudomonas palustris for the obligate reduction of n-butyrate to n-butanol
Source: Biotechnol Biofuels. 2017 Jul 11;10:178. doi: 10.1186/s13068-017-0864-3 (PMC5504763; doi:10.1186/s13068-017-0864-3)
Supplement: Supplementary file 1 — Additional file 1. Growth with butyrate, containing Figure S1. [file 13068_2017_864_MOESM1_ESM.docx]

**1. *n*-Butyrate toxicity experiment**


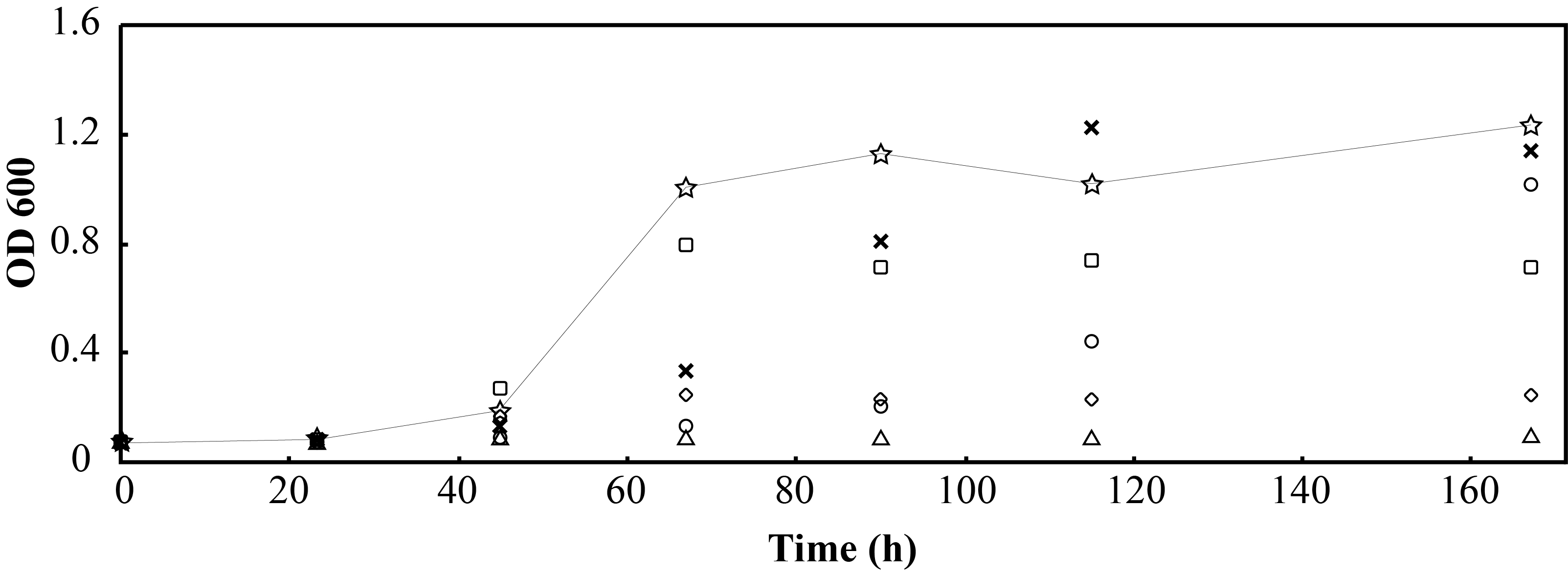


**Figure S1.** Growth curves of *R. palustris* CGA009 in anaerobic serum bottles with RM media containing a range of *n*-butyrate concentrations: 0 g/L (triangle), 0.1 g/L (diamond), 0.5 g/L (square), 1.0 g/L (star), 2.5 g/L (cross), 5.0 g/L (circle).
