# Supplementary material for: Metabolic engineering of Rhodopseudomonas palustris for the obligate reduction of n-butyrate to n-butanol
Source: Biotechnol Biofuels. 2017 Jul 11;10:178. doi: 10.1186/s13068-017-0864-3 (PMC5504763; doi:10.1186/s13068-017-0864-3)
Supplement: Supplementary file 2 — Additional file 2. Sequence. [file 13068_2017_864_MOESM2_ESM.docx]

**2. Sequence**

**Sequence of *adhE2* _opti_**: Restriction sites for cloning are underlined. Coding sequence of gene indicated by Capital letters. 6X His tag used for Western blot detection is indicated in red.

ctcgaggaaataaggcaacgtacgaaggaggtaaataATGAAGGTCACCAACCAGAAGGAACTGAAGCAGAAGCTGAACGAGCTGCGGGAAGCCCAGAAGAAGTTCGCCACCTACACCCAGGAGCAGGTGGACAAGATCTTCAAGCAGTGCGCCATCGCGGCCGCGAAGGAACGCATCAACCTGGCCAAGCTGGCGGTGGAGGAAACCGGCATCGGCCTGGTCGAGGACAAGATCATCAAGAACCACTTCGCCGCGGAATACATCTATAACAAGTACAAGAACGAGAAGACCTGCGGCATCATCGACCATGACGACAGCCTGGGCATCACCAAGGTGGCCGAGCCGATCGGCATCGTGGCCGCGATCGTCCCGACCACCAACCCGACCTCGACCGCGATCTTCAAGAGCCTGATCTCCCTGAAGACCCGCAACGCGATCTTCTTCAGCCCGCACCCGCGGGCCAAGAAGTCCACCATCGCCGCGGCCAAGCTGATCCTGGACGCGGCCGTCAAGGCGGGCGCCCCGAAGAACATCATCGGCTGGATCGACGAACCGTCCATCGAGCTGTCGCAGGACCTGATGTCGGAGGCCGACATCATCCTGGCGACCGGCGGCCCGAGCATGGTGAAGGCGGCCTACTCGTCGGGCAAGCCGGCCATCGGCGTCGGCGCGGGCAACACCCCGGCCATCATCGACGAGTCCGCGGACATCGACATGGCCGTGTCCTCGATCATCCTGTCGAAGACCTATGACAACGGCGTGATCTGCGCCTCGGAACAGAGCATCCTGGTCATGAACAGCATCTACGAGAAGGTGAAGGAGGAATTCGTCAAGCGCGGCTCGTATATCCTGAACCAGAACGAAATCGCCAAGATCAAGGAGACCATGTTCAAGAACGGCGCCATCAACGCGGACATCGTGGGCAAGAGCGCGTACATCATCGCCAAGATGGCGGGCATCGAAGTCCCGCAGACCACCAAGATCCTGATCGGCGAGGTGCAGAGCGTCGAAAAGTCCGAGCTGTTCTCGCACGAGAAGCTGTCGCCGGTGCTGGCGATGTACAAGGTCAAGGACTTCGACGAAGCCCTGAAGAAGGCGCAGCGCCTGATCGAGCTGGGCGGCTCGGGCCATACCAGCTCCCTGTATATCGACAGCCAGAACAACAAGGACAAGGTGAAGGAGTTCGGCCTGGCCATGAAGACCTCGCGGACCTTCATCAACATGCCGTCGAGCCAGGGCGCGTCCGGCGACCTGTATAACTTCGCGATCGCCCCGTCGTTCACCCTGGGCTGCGGCACCTGGGGCGGCAACTCCGTGTCGCAGAACGTCGAGCCGAAGCATCTGCTGAACATCAAGTCGGTGGCCGAACGCCGGGAGAACATGCTGTGGTTCAAGGTCCCGCAGAAGATCTACTTCAAGTATGGCTGCCTGCGCTTCGCCCTGAAGGAACTGAAGGACATGAACAAGAAGCGGGCGTTCATCGTCACCGACAAGGACCTGTTCAAGCTGGGCTACGTGAACAAGATCACCAAGGTCCTGGACGAGATCGACATCAAGTATTCGATCTTCACCGACATCAAGTCGGACCCGACCATCGACAGCGTGAAGAAGGGCGCGAAGGAAATGCTGAACTTCGAGCCGGACACCATCATCAGCATCGGCGGCGGCTCCCCGATGGACGCGGCCAAGGTCATGCACCTGCTGTACGAGTATCCGGAGGCGGAAATCGAGAACCTGGCCATCAACTTCATGGACATCCGCAAGCGGATCTGCAACTTCCCGAAGCTGGGCACCAAGGCCATCAGCGTGGCGATCCCGACCACCGCCGGCACCGGCTCCGAAGCCACCCCGTTCGCGGTCATCACCAACGACGAGACCGGCATGAAGTACCCGCTGACCAGCTATGAACTGACCCCGAACATGGCGATCATCGACACCGAGCTGATGCTGAACATGCCGCGCAAGCTGACCGCGGCCACCGGCATCGACGCCCTGGTGCATGCCATCGAAGCGTACGTGTCGGTCATGGCGACCGACTATACCGACGAGCTGGCCCTGCGCGCGATCAAGATGATCTTCAAGTACCTGCCGCGTGCGTATAAGAACGGCACCAACGACATCGAAGCGCGGGAGAAGATGGCGCACGCCAGCAACATCGCGGGCATGGCGTTCGCCAACGCGTTCCTGGGCGTGTGCCACTCGATGGCGCATAAGCTGGGCGCCATGCACCATGTCCCGCATGGCATCGCCTGCGCGGTGCTGATCGAGGAAGTCATCAAGTACAACGCGACCGACTGCCCGACCAAGCAGACCGCCTTCCCGCAGTATAAGTCGCCGAACGCGAAGCGCAAGTACGCCGAAATCGCGGAGTATCTGAACCTGAAGGGCACCAGCGACACCGAAAAGGTCACCGCCCTGATCGAGGCGATCTCCAAGCTGAAGATCGACCTGTCCATCCCGCAGAACATCTCGGCGGCCGGCATCAACAAGAAGGACTTCTACAACACCCTGGACAAGATGTCGGAGCTGGCGTTCGACGACCAGTGCACCACCGCCAACCCGCGCTATCCGCTGATCTCCGAGCTGAAGGACATCTACATCAAGTCGTTCCACCACCATCACCATCACTAAtctaga
