# Supplementary material for: Metabolic engineering of Rhodopseudomonas palustris for the obligate reduction of n-butyrate to n-butanol
Source: Biotechnol Biofuels. 2017 Jul 11;10:178. doi: 10.1186/s13068-017-0864-3 (PMC5504763; doi:10.1186/s13068-017-0864-3)
Supplement: Supplementary file 3 — Additional file 3. Fluorescence, containing Figure S2. [file 13068_2017_864_MOESM3_ESM.docx]

**3. Fluorescence**


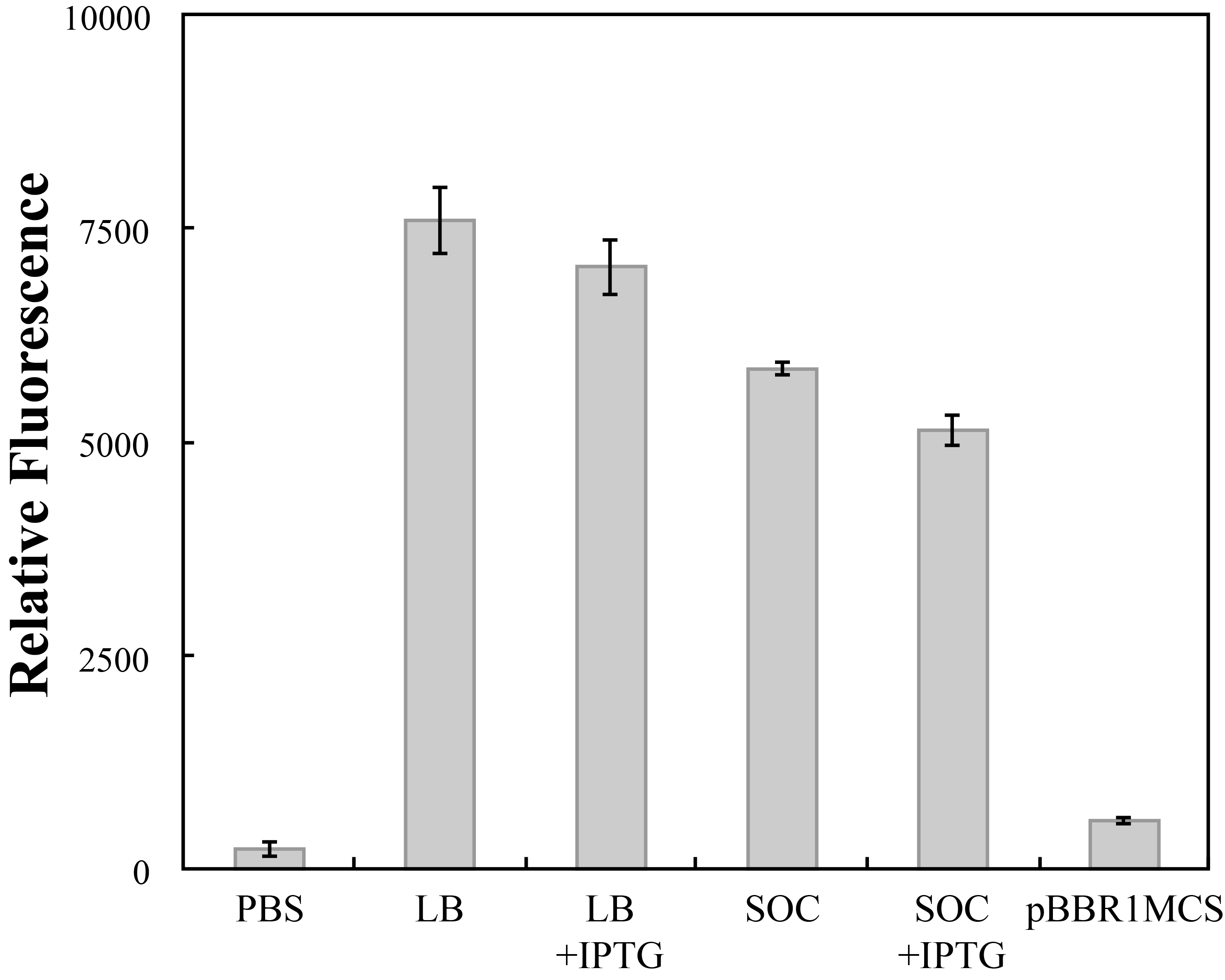


**Figure S2.** Response of pBBR1MCS-2 *lacp GFPmut2* expression vector to IPTG induction and catabolite repression in *R. palustris* CGA009*.* Fluorescence is expressed as relative fluorescence per 1 unit of OD_600_.
