# Supplementary material for: Metabolic engineering of Rhodopseudomonas palustris for the obligate reduction of n-butyrate to n-butanol
Source: Biotechnol Biofuels. 2017 Jul 11;10:178. doi: 10.1186/s13068-017-0864-3 (PMC5504763; doi:10.1186/s13068-017-0864-3)
Supplement: Supplementary file 4 — Additional file 4. Structure, containing Figure S3. [file 13068_2017_864_MOESM4_ESM.docx]

**4. Structures**


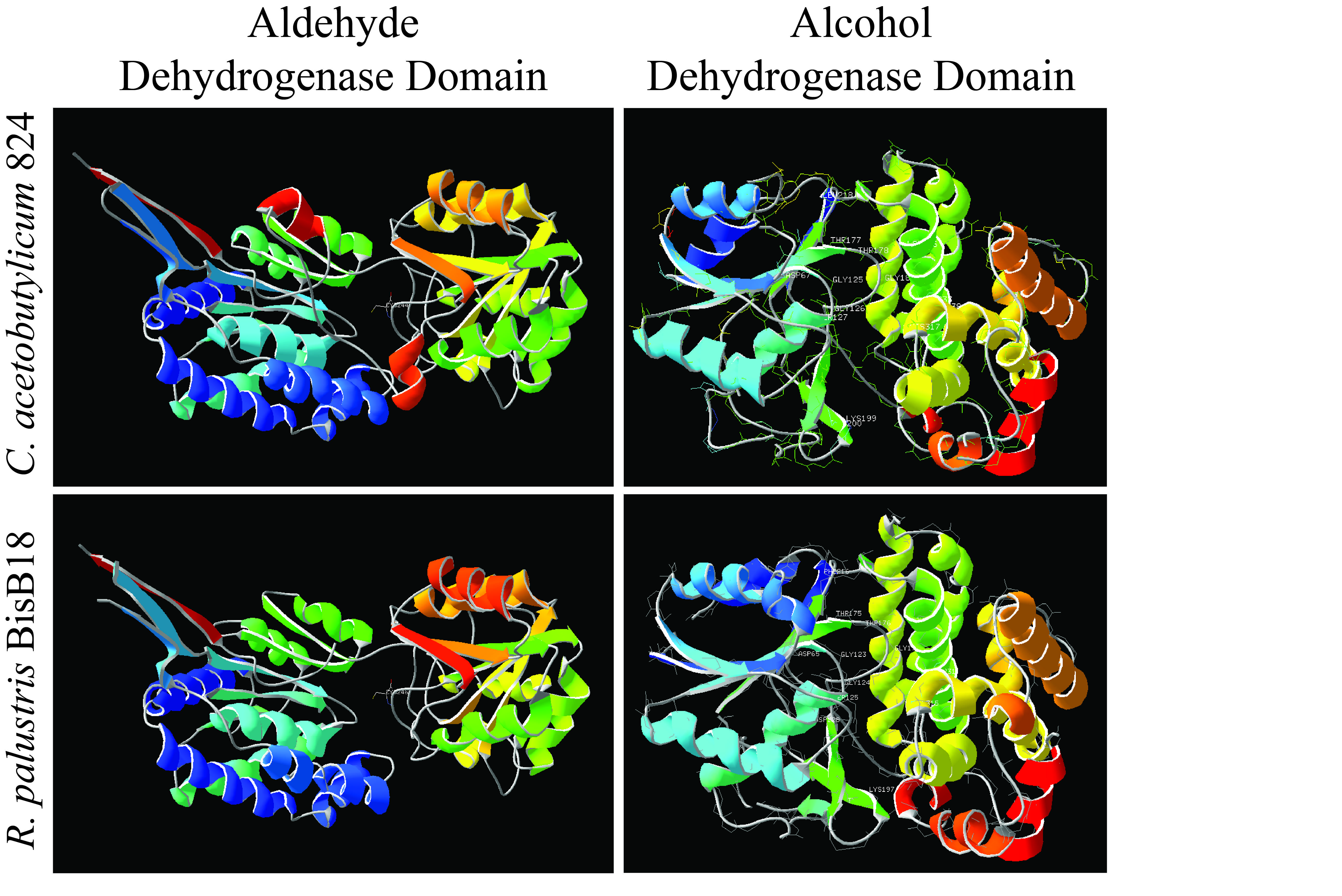


**Figure S3.** Predicted structures of individual aldehyde and alcohol domains for AdhE2 _824_ from *C. acetobutylicum* and AdhE _BisB18_ from *R. palustris*. Domains were modeled separately with Swiss PDBViewer using pdb3m7C (e^-132^ structural similarity) as a scaffold for modeling the alcohol dehydrogenase domain and pdb3zdr (e^-114^ structural similarity) as a scaffold for the alcohol domains.
