# Supplementary material for: Metabolic engineering of Rhodopseudomonas palustris for the obligate reduction of n-butyrate to n-butanol
Source: Biotechnol Biofuels. 2017 Jul 11;10:178. doi: 10.1186/s13068-017-0864-3 (PMC5504763; doi:10.1186/s13068-017-0864-3)
Supplement: Supplementary file 5 — Additional file 5. Codons, containing Figure S4. [file 13068_2017_864_MOESM5_ESM.docx]

**5. Codons**


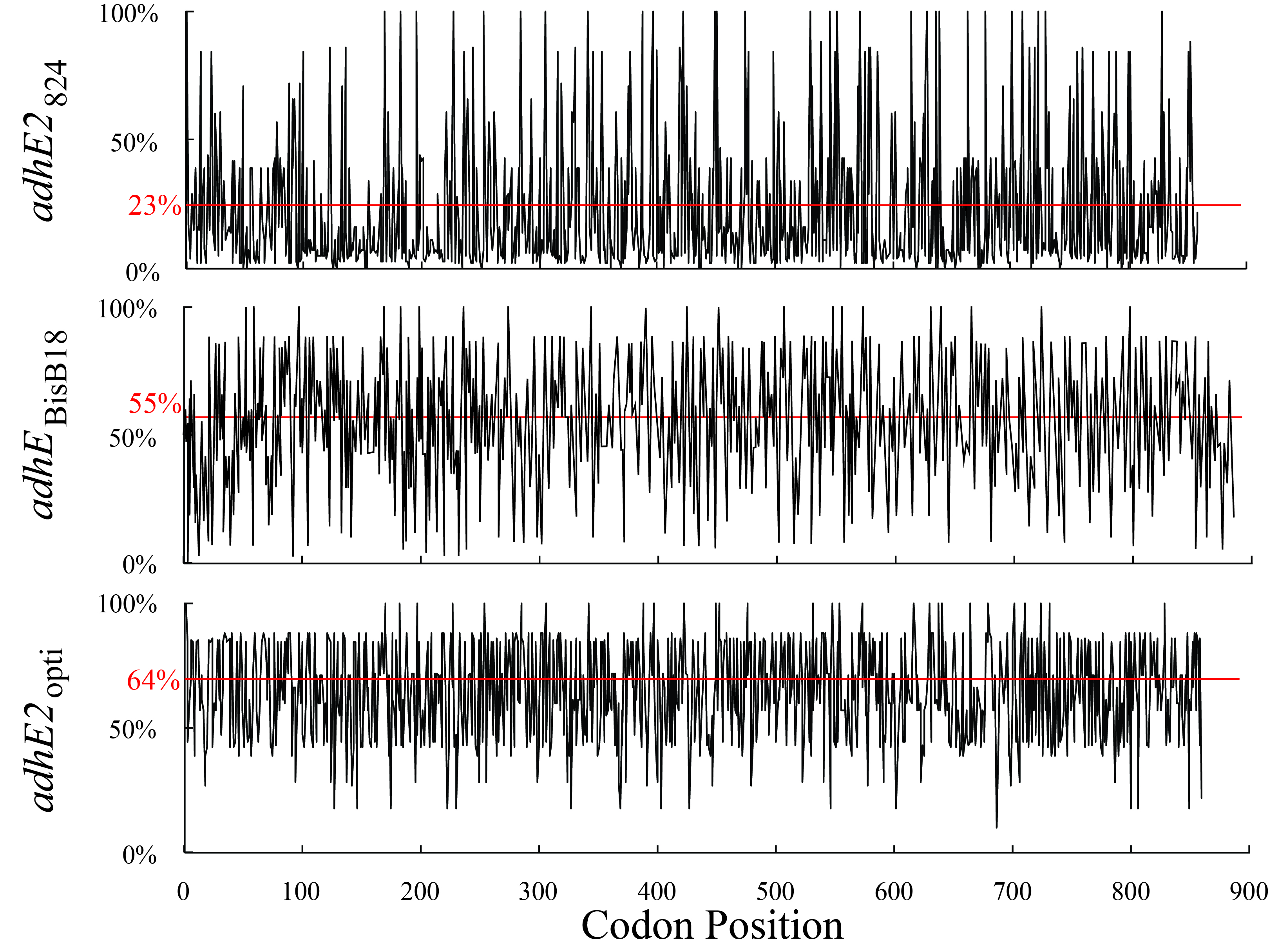


**Figure S4**. Codon utilization frequency for each codon position appearing in the three candidate enzymes for butyryl-CoA to butanol conversion: top panel) *adhE2* _824_ from *C. acetobutylicum*; middle panel) *adhE* _BisB18_ from *R. palustris*; and lower panel) *adhE2* _opti_.
