# Supplementary material for: Metabolic engineering of Rhodopseudomonas palustris for the obligate reduction of n-butyrate to n-butanol
Source: Biotechnol Biofuels. 2017 Jul 11;10:178. doi: 10.1186/s13068-017-0864-3 (PMC5504763; doi:10.1186/s13068-017-0864-3)
Supplement: Supplementary file 6 — Additional file 6. Codons and GC content, containing Table S1. [file 13068_2017_864_MOESM6_ESM.docx]

**6. Codons and GC content**

**Table S1.** Codon utilization frequency in *R. palustris*, GC content, and rare codon percentage for the three alcohol/aldehyde dehydrogenase genes used in this study.

| **Gene** | **Codon utilization frequency** | **GC content** | **Rare codon percentage** |
| --- | --- | --- | --- |
| *adhE2* _824_ | 23% | 30% | 47% |
| *adhE* _BisB18_ | 55% | 63% | 5% |
| *adhE2* _opti_ | 64% | 65% | 0% |
