# Supplementary material for: Metabolic engineering of Rhodopseudomonas palustris for the obligate reduction of n-butyrate to n-butanol
Source: Biotechnol Biofuels. 2017 Jul 11;10:178. doi: 10.1186/s13068-017-0864-3 (PMC5504763; doi:10.1186/s13068-017-0864-3)
Supplement: Supplementary file 7 — Additional file 7. Growth with butanol, containing Figure S5. [file 13068_2017_864_MOESM7_ESM.docx]

**7. Growth with butanol**


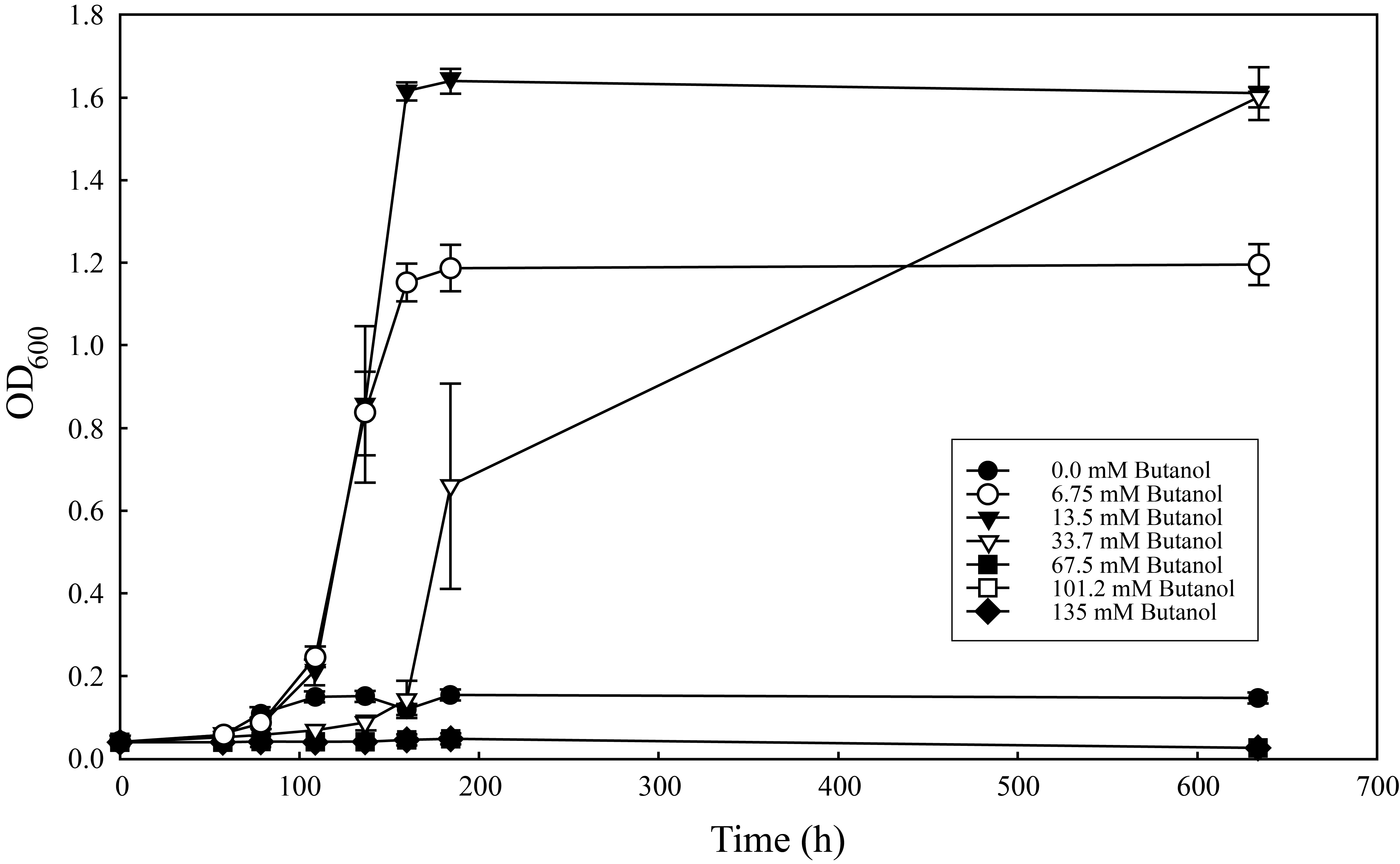


**Figure S5**. Growth of *R. palustris* CGA009 with butanol as a substrate in triplicate bottles*.* A minor lag phase was observed for 33.7 mM butanol, but maximum growth was ultimately achieved. A low growth rate was observed after a much longer lag phase for 67.5 and 101.2 mM butanol (data not shown). No growth was observed for 0 mM butanol due to the absence of substrate, and no growth was observed for the 135 mM butanol due to toxicity.
