# Supplementary material for: Metabolic engineering of Rhodopseudomonas palustris for the obligate reduction of n-butyrate to n-butanol
Source: Biotechnol Biofuels. 2017 Jul 11;10:178. doi: 10.1186/s13068-017-0864-3 (PMC5504763; doi:10.1186/s13068-017-0864-3)
Supplement: Supplementary file 8 — Additional file 8. Growth with product removal, containing Figure S6. [file 13068_2017_864_MOESM8_ESM.docx]

**8. Growth with product removal**


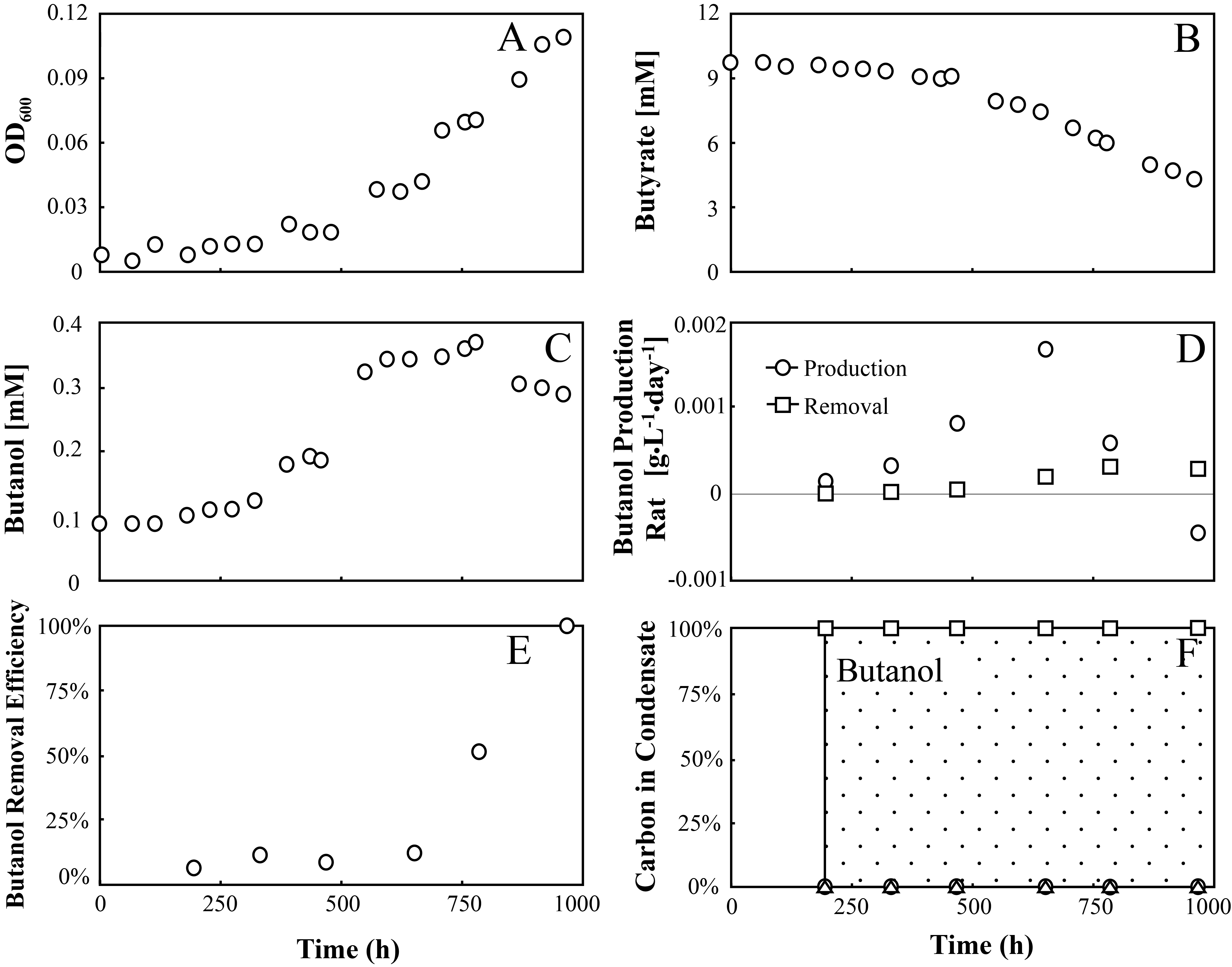


**Figure S6.** Performance of engineered *R. palustris* pBBR1MCS-2 *lacp adhE2* _opti_ without HCO_3_^-^ in a batch bioreactor with product removal: (**A**) growth; (**B**) butyrate concentration in the bioreactor broth; (**C**) butanol concentration in the bioreactor broth; (**D**) volumetric butanol production rate; this production rate includes both butanol measured in the bioreactor and collected in the condensers; (**E**) butanol removal efficiency; this efficiency was calculated by dividing the butanol in the condenser by the total butanol produced; (**F**) percentage of carbon in the condensate as butanol.
